# Impact of Cancer Subtype and Cancer Therapy Exposures on SARS‐CoV‐2 Outcomes in the Omicron and Subvariant Era

**DOI:** 10.1002/cam4.71082

**Published:** 2025-07-22

**Authors:** Katelyn M. Atkins, Minhao Wang, Katrina D. Silos, Sandy Y. Joung, Asneh Singh, Olivia Peony, Jordan O. Gasho, Yeran Lee, Kenia Gastelum, Beatrice Alessandra Filart, Alan C. Kwan, Marilyn Mendez, Yunxian Liu, Patrick Belen, John C. Prostko, Edwin C. Frias, Joseph E. Ebinger, Sonia Sharma, Kimia Sobhani, Susan Cheng

**Affiliations:** ^1^ Department of Radiation Oncology Cedars‐Sinai Medical Center Los Angeles California USA; ^2^ Department of Cardiology Smidt Heart Institute, Cedars‐Sinai Medical Center Los Angeles California USA; ^3^ Applied Research and Technology Abbott Diagnostics Abbott Park Illinois USA; ^4^ La Jolla Institute of Immunology La Jolla California USA; ^5^ Department of Pathology and Laboratory Medicine Cedars‐Sinai Medical Center Los Angeles California USA

**Keywords:** B‐cell‐targeted therapy, hematologic cancer, SARS‐CoV‐2

## Abstract

**Introduction:**

Amidst highly transmissible SARS‐CoV‐2 variants that continue to circulate in the community, individuals with cancer exhibit variations in immunity and susceptibility for reasons that remain poorly understood.

**Methods:**

In a longitudinal cohort study with ongoing SARS‐CoV‐2 serological and outcomes surveillance, we examined adults receiving cancer treatment (cases, *n* = 229) or who were free of cancer and other major comorbidities (controls, *n* = 800), prior to the Omicron era onset and onwards (September 24, 2021–March 10, 2024). The main outcomes were longitudinal SARS‐CoV‐2 anti‐spike receptor binding domain IgG (IgG‐SRBD) antibody response and Omicron and subvariant infection frequency and severity.

**Results:**

Among the 229 participants with cancer (age 66 ± 12 years, 51% female), the most prevalent subtypes included nonmelanoma skin (23%), breast (20%), and hematologic (18%). In mixed‐effects linear models, hematologic cancer and B‐cell targeted agents were associated with reduced longitudinal IgG‐SRBD response (*p* < 0.05). In multivariable regression analyses, hematologic cancer (*p* = 0.037) and B‐cell targeted agents (*p* = 0.030) were associated with increased frequency of new infections. The frequency of new infections resulting in moderate illness was increased in patients with active/recent cancer treatment (44%) versus healthy controls (10%; *p* < 0.001); there were no severe or critical infections. Patients with hematologic, breast, prostate, or skin cancer (*p* < 0.01), treated with local therapy (odds ratio [OR] 1.82; 95% confidence interval [CI] 1.05–3.15; *p* = 0.032), B‐cell targeted therapy (OR 4.81; 95% CI 1.78–12.93; *p* = 0.002), or small molecule agents (OR 2.34; 95% CI 1.05–5.23; *p* = 0.037) were associated with increased infection severity.

**Conclusions:**

Individuals with hematologic cancer or exposed to B‐cell‐targeted therapy had reduced humoral immunity and more frequent and severe infections. Active breast, prostate, or skin cancer, or treatment with local therapy or small molecule agents had elevated risk for more severe, but not more frequent, infections. Despite overall low rates of infection associated with lower respiratory disease, certain higher‐risk cancer patients may benefit from further protective measures.

## 1 Introduction

Currently, in the postpandemic era, highly transmissible SARS‐CoV‐2 variants continue to evolve and circulate in the community. Individuals with cancer remain at high risk for infection and related sequelae, at least partly due to their relatively immunocompromised status. Notwithstanding the broad heterogeneity represented by persons with cancer, populations of cancer compared to noncancer patients are reported to have a generally higher incidence of SARS‐CoV‐2 infections [1, 2, 3, 4] and an associated greater risk of severe illness and death [5, 6, 7, 8]. In particular, SARS‐CoV‐2 mortality risk appears highest in persons with hematologic malignancies [9] and in patients actively being treated with chemotherapy [10], although the prior available data remain limited and inconsistent regarding distinct cancer types and anti‐cancer treatments in relation to COVID‐19 outcomes [9, 11, 12, 13]. Importantly, most prior studies were conducted in the setting of the original and early SARS‐CoV‐2 variants, resulting in relatively scant information regarding relevant SARS‐CoV‐2 outcomes in the current era which is dominated by Omicron and its shifting subvariants, the most prevalent of which is now JN.1, a subvariant that is closely related to the BA.2.86 lineage of Omicron. JN.1 accounted for an estimated 83%–88% of circulating variants in the U.S. by January 2024. Given the intrinsic demographic and clinical diversity of cancer patient populations, in addition to the range of the treatments they receive including multi‐modality treatments, a detailed understanding of the cancer‐specific risks associated with SARS‐CoV‐2 susceptibility and outcomes in the Omicron and subvariant era is needed. To this end, we performed a comprehensive longitudinal cohort study of patients with cancer who have been under continued clinical surveillance from prior to the emergence of Omicron and its subvariants. We sought to identify the specific cancer subtypes and treatment exposures associated with durable humoral immunity as well as new‐onset SARS‐CoV‐2 infection frequency and severity.

## 2 Methods

### 2.1 Study Cohort

We enrolled into a longitudinal SARS‐CoV‐2 surveillance study a diverse population of adult patients and employees at Cedars‐Sinai Medical Center in Los Angeles, California [14]. All participants completed baseline and serial electronic surveys on medical history, SARS‐CoV‐2 exposures (i.e., number and timing of infections and vaccines), and symptoms, for which several SARS‐CoV‐2 outcomes studies have been reported [14, 15, 16]. Participants are invited to provide up to monthly blood draws for SARS‐CoV‐2 serological assays. The presence or absence of self‐reported comorbidities was verified via review of the electronic medical record. Of the 10,640 participants enrolled in the source study, we included 1029 whose enrollment spanned from September 24, 2021, to March 10, 2024: 800 referent healthy participants (free of cancer and other major comorbidities) and 229 patients with cancer whose primary cancer treatment overlapped the SARS‐CoV‐2 pandemic onset, defined as on or after March 1, 2020. This was a sample size of convenience, as all adult (age ≥ 18 years) patients with a self‐reported history of cancer were screened for eligibility (primary cancer treatment during and/or overlapping the SARS‐CoV‐2 pandemic), and all eligible referent healthy participants (defined as without cancer or other major medical comorbidity) were included. Of these, 833 had at least one blood draw for SARS‐CoV‐2 anti‐spike antibody (IgG‐SRBD) and anti‐nucleocapsid antibody (IgG‐N) analysis. We excluded from analyses individuals who were missing key covariate data (*n* = 177), or whose primary cancer treatment occurred prior to the pandemic onset (*n* = 31) (Figure S1). All participants provided written informed consent. This study was approved by the Cedars‐Sinai Medical Center institutional review board. This report follows the Strengthening the Reporting of Observational Studies in Epidemiology (STROBE) reporting guideline for observational studies.

### 2.2 Cancer Assessment

Cancer subtype and treatment details were collected through comprehensive manual EHR review (e.g., pathology reports, operative reports, oncology notes, imaging reports). Patients with any stage malignancy and with anti‐cancer treatment occurring since the onset of the pandemic (defined as March 1, 2020) were included, except for patients with breast or prostate cancer who had primary surgery *prior* to pandemic onset and were on hormone therapy alone (*n* = 31); these latter patients were excluded from analyses. Date(s) and subtype(s) of all anti‐cancer treatments were collected. Local therapy included surgery, local excisions, ablative procedures (i.e., intravascular, percutaneous), and/or topical treatments. Systemic therapies were analyzed and categorized into the following groups: cytotoxic chemotherapy (including stem cell transplant), B‐cell targeted therapies (anti‐CD20, anti‐CD38 monoclonal antibodies [mAbs]), biologic agents (including non‐anti‐CD20/CD38 mAbs), immune checkpoint inhibitors, small molecule targeted agents, and hormonal therapies. Radiotherapy (RT) included external beam radiotherapy in addition to brachytherapy and radioembolization.

### 2.3 Serological and Infection Assessments

Serological assays testing for antibodies to the receptor binding domain of the S1 subunit of the viral spike protein (IgG‐SRBD) and the viral nucleocapsid protein (IgG‐N) were performed using the Abbott Architect immunoassay (Abbott Laboratories, Abbott Park, IL). An IgG‐N index value of ≥ 1.4 was considered to represent previous SARS‐CoV‐2 infection, and any subsequent new infection was determined based on change in IgG‐N index level from < 1.2 to ≥ 1.4 [14, 17]. Omicron infections were determined as those that occurred after the start of the Omicron variant surge (December 15, 2021) in the Southern California region [18]. SARS‐CoV‐2 primary infection data were collected from the REDCap electronic survey database (Vanderbilt University) and from the EMR using algorithm‐based scripts. For SARS‐CoV‐2 events that were not captured in our EMR, manual verification was performed by trained study staff. Self‐reported infection events were verified using outside polymerase chain reaction (PCR) records or other testing. Self‐reported vaccination events were confirmed with vaccination cards or other external records. All manual verification protocols used standardized data collection instruments and were performed in a batch manner for the overall study, thus blinding study staff to hypotheses for specific cohorts (including the present study). SARS‐CoV‐2 infection severity was assessed based on associated symptoms, evidence of lower respiratory disease, oxygen saturation measured by pulse oximetry (SpO_2_) on room air, ratio of arterial partial pressure of oxygen to fraction of inspired oxygen (PaO2/FiO2), respiratory rate, and lung infiltrates [19, 20]; these data were captured via self‐reported surveys, review of medical records, phone and email logs, and health update questionnaires conducted at each study visit. Mild illness was defined as having COVID‐19 symptoms without shortness of breath, dyspnea, or abnormal chest imaging; moderate illness included evidence of lower respiratory disease with SpO_2_ ≥ 94%; severe illness included evidence of lower respiratory disease SpO_2_ < 94%, PaO_2_/FiO_2_ < 300 mmHg, a respiratory rate > 30 breaths/min, or lung infiltrates > 50%; critical illness was defined as respiratory failure, septic shock, or multiple organ dysfunction [19, 20].

### 2.4 Statistical Analyses

The distribution of baseline clinical cohort characteristics was evaluated using descriptive statistics. Continuous variables were compared using the unpaired, two‐tailed *t* test (or Kruskal‐Wallis rank sum for nonparametric continuous variables) and categorical variables were compared using the Chi‐Squared test. To evaluate the pattern and predictors of the longitudinal IgG‐SRBD response over time, a mixed‐effects linear model was generated with each participant's repeated IgG‐SRBD measures treated as random effects to estimate the mean log_10_(IgG‐SRBD) associated with time. Model output was graphically displayed using natural cubic splines, indexed by time from their initial vaccine series or last booster. Multivariable Poisson regression was performed to examine the association between cancer and cancer treatment exposures with the total number of Omicron (and subvariant) infections. For cancer treatment exposures, local therapy only was treated as a distinct variable, and exposure to other treatments (e.g., biologics) was each treated as a binary (any versus never) for each type of treatment. Due to collinearity between cancer subtypes and treatment exposures, separate models for cancer subtypes versus cancer treatments were performed. Multivariable ordinal logistic regression was performed to examine the association between cancer and cancer treatment exposures with the severity of Omicron and subvariant infection. All statistical analyses were performed using R (v4.2.1) with a two‐tailed *p* < 0.05 considered statistically significant.

## 3 Results

### 3.1 Cohort Characteristics

Participants in the cancer cohort were significantly older (mean 65.5 ± 11.9 years) than the healthy referent cohort (41.0 ± 11.4 years; *p* < 0.001) (Table 1). Further, those in the cancer cohort compared to the healthy referent cohort were more likely to be male (49.3% vs. 39.0%; *p* = 0.006), self‐identify as white race (85.6% vs. 48.2%; *p* < 0.001), and have participated in more study blood draws (mean 6.0 vs. 4.3; *p* < 0.001). Participants in the cancer cohort were less likely than their counterparts to be a healthcare employee (20.1% vs. 72.1%; *p* < 0.001) or to have had any SARS‐CoV‐2 infection prior to their first vaccination (6.1% vs. 11.1%; *p* = 0.035). There were no between‐cohort differences in the average number of SARS‐CoV‐2 infections or in the total cohort follow‐up time contributed to the study (*p* > 0.05).

**TABLE 1:** Demographic and clinical characteristics, by cancer status.

Characteristics	Overall	Cancer	Healthy	*p*
	(*n* = 1029)	(*n* = 229)	(*n* = 800)	
Age, years, mean (SD)	46.47 (15.40)	65.53 (11.92)	41.01 (11.43)	< 0.001
Sex, Male, *n* (%)	425 (41.3)	113 (49.3)	312 (39.0)	0.006
Hispanic ethnicity, *n* (%)	153 (14.9)	17 (7.4)	136 (17.0)	< 0.001
Race, *n* (%)				< 0.001
American Indian/Alaska Native	6 (0.6)	1 (0.4)	5 (0.6)	
Asian	273 (26.5)	15 (6.6)	258 (32.2)	
Black or African American	44 (4.3)	7 (3.1)	37 (4.6)	
Multiple	47 (4.6)	3 (1.3)	44 (5.5)	
Native Hawaiian or other Pacific Islander	17 (1.7)	1 (0.4)	16 (2.0)	
Other	60 (5.8)	6 (2.6)	54 (6.8)	
White	582 (56.6)	196 (85.6)	386 (48.2)	
**Comorbidities**				
Obesity, *n* (%)	141 (13.7)	45 (19.7)	96 (12.0)	0.004
Hypertension, *n* (%)	103 (10.0)	103 (45.0)	0 (0.0)	—
Diabetes, *n* (%)	36 (3.5)	36 (15.7)	0 (0.0)	—
Organ transplant, *n* (%)	62 (6.0)	62 (27.1)	0 (0.0)	—
Heart disease^a^, *n* (%)	76 (7.4)	76 (33.2)	0 (0.0)	—
Autoimmune disease, *n* (%)	52 (5.1)	52 (22.7)	0 (0.0)	—
Liver disease, *n* (%)	93 (9.0)	93 (40.6)	0 (0.0)	—
Kidney disease, *n* (%)	72 (7.0)	72 (31.4)	0 (0.0)	—
Any cardiovascular or pulmonary vascular disease^b^, *n* (%)	156 (15.2)	156 (68.1)	0 (0.0)	—
Neurological disease, *n* (%)	50 (4.9)	50 (21.8)	0 (0.0)	—
**Cancer details**				
Metastatic disease, *n* (%)	21 (2.0)	21 (9.2)	0 (0.0)	—
Cancer Type, *n* (%)				—
No reported cancer or other comorbidities	800 (77.7)	0 (0.0)	800 (100.0)	
Breast	46 (4.5)	46 (20.1)	0 (0.0)	
Hematologic	41 (4.0)	41 (17.9)	0 (0.0)	
Nonmelanoma skin	53 (5.2)	53 (23.1)	0 (0.0)	
Prostate	22 (2.1)	22 (9.6)	0 (0.0)	
Other solid	67 (6.5)	67 (29.3)	0 (0.0)	
Cancer Treatment				
Local therapy/surgery (only), *n* (%)	101 (9.8)	101 (44.1)	0 (0.0)	—
Radiotherapy (any), *n* (%)	69 (6.7)	69 (30.1)	0 (0.0)	—
Cytotoxic chemotherapy (any), *n* (%)	57 (5.5)	57 (24.9)	0 (0.0)	—
B‐cell targeted agents (any), *n* (%)	26 (2.5)	26 (11.4)	0 (0.0)	—
Biologics (non‐B‐cell targeted) (any), *n* (%)	13 (1.3)	13 (5.7)	0 (0.0)	—
Immune checkpoint inhibitors (any), *n* (%)	11 (1.1)	11 (4.8)	0 (0.0)	—
Small molecule targeted agents (any), *n* (%)	34 (3.3)	34 (14.8)	0 (0.0)	—
Hormonal therapy (any), *n* (%)	38 (3.7)	38 (16.6)	0 (0.0)	—
Total cancer treatment length, year, mean (SD)	0.92 (5.17)	4.53 (10.78)	0.00 (0.00)	—
**SARS‐CoV‐2 details**				
Any SARS‐CoV‐2 infection prior to dose 1 vaccination, *n* (%)	103 (10.0)	14 (6.1)	89 (11.1)	0.035
No. of SARS‐CoV‐2 booster(s), mean (SD)	2.05 (1.24)	3.01 (1.42)	1.77 (1.03)	< 0.001
Total follow‐up time, month, mean (SD)	23.14 (6.22)	23.62 (4.88)	23.01 (6.54)	0.19
Healthcare employee, *n* (%)	623 (60.5)	46 (20.1)	577 (72.1)	< 0.001
No. of SARS‐CoV‐2 infection, mean (SD)	0.79 (0.81)	0.86 (0.83)	0.78 (0.80)	0.16
No. of SARS‐CoV‐2 infection, *n* (%)				0.22
0	423 (41.1)	84 (36.7)	339 (42.4)	
1	430 (41.8)	103 (45.0)	327 (40.9)	
2	145 (14.1)	34 (14.8)	111 (13.9)	
3	28 (2.7)	6 (2.6)	22 (2.8)	
≥ 4	3 (0.3)	2 (0.9)	1 (0.1)	
No. of new‐onset Omicron SARS‐CoV‐2 infection, mean (SD)	0.61 (0.70)	0.74 (0.72)	0.57 (0.68)	0.001
No. of new‐onset Omicron SARS‐CoV‐2 infection, *n* (%)				0.008
0	522 (50.7)	94 (41.0)	428 (53.5)	
1	396 (38.5)	103 (45.0)	293 (36.6)	
2	103 (10.0)	29 (12.7)	74 (9.2)	
3	8 (0.8)	3 (1.3)	5 (0.6)	
Total tixagevimab‐cilgavimab dosage, g, mean (SD)	0.04 (0.19)	0.16 (0.37)	0.00 (0.00)	—
Total tixagevimab‐cilgavimab dose(s), mean (SD)	0.07 (0.39)	0.33 (0.77)	0.00 (0.00)	—
Total tixagevimab‐cilgavimab dose(s), *n* (%)				—
0	986 (95.8)	186 (81.2)	800 (100.0)	
1	19 (1.8)	19 (8.3)	0 (0.0)	
2	16 (1.6)	16 (7.0)	0 (0.0)	
3	7 (0.7)	7 (3.1)	0 (0.0)	
≥ 4	1 (0.1)	1 (0.4)	0 (0.0)	
No. of blood draw encounters, mean (SD)	4.63 (5.23)	5.96 (5.13)	4.26 (5.20)	< 0.001
No. of blood draw encounters, log10, mean (SD)	0.40 (0.49)	0.59 (0.43)	0.35 (0.49)	< 0.001

^a^ Heart disease refers to coronary heart disease (e.g., myocardial infarction, ischemic heart disease), ischemic heart failure, or atrial fibrillation or flutter.

^b^ Any cardiovascular or pulmonary vascular disease refers to any heart disease (defined above), in addition to any non‐atrial fibrillation or flutter arrhythmias, cerebrovascular disease, peripheral vascular disease, or pulmonary vascular disorders.

Among participants with cancer treatment during the pandemic, the most common cancer subtypes were nonmelanoma skin cancer (23.1%), breast cancer (20.1%), hematologic cancers (17.9%), and prostate cancer (9.6%). The remaining lower frequency solid malignancies were grouped into an “other” category (29.3%) of which liver, renal, melanoma, colorectal, and lung were most common (Table S1). Metastatic disease was present in 9.2% of patients. Nearly half of the participants were treated with local therapy only (44.1%; most commonly surgery [*n* = 143]), while 30.1% received radiotherapy (most commonly external beam [*n* = 62] to the breast [*n* = 26] or abdomen/pelvis [*n* = 23]), 24.9% cytotoxic chemotherapy (most commonly platinum‐based regimens [*n* = 19]), 14.8% small molecule targeted agents (most commonly proteasome inhibitors [*n* = 16] and tyrosine kinase inhibitors [*n* = 9]), 11.4% B‐cell targeted agents (most commonly daratumumab/anti‐CD38 [*n* = 14] and rituximab/anti‐CD20 [*n* = 12]), 5.7% biologics (non‐B‐cell targeted) agents (most commonly human epidermal growth factor receptor (HER2) targeted [*n* = 7]), 4.8% with immune checkpoint inhibitors (most commonly pembrolizumab [*n* = 5]), and 16.6% with hormonal therapy (most commonly aromatase inhibitors [*n* = 19], tamoxifen [*n* = 10], and leuprolide [*n* = 9]; Table 1, and Table S2 for detailed classification of all cancer treatment exposures).

### 3.2 Longitudinal IgG‐SRBD Antibody Response

**FIGURE 1:**
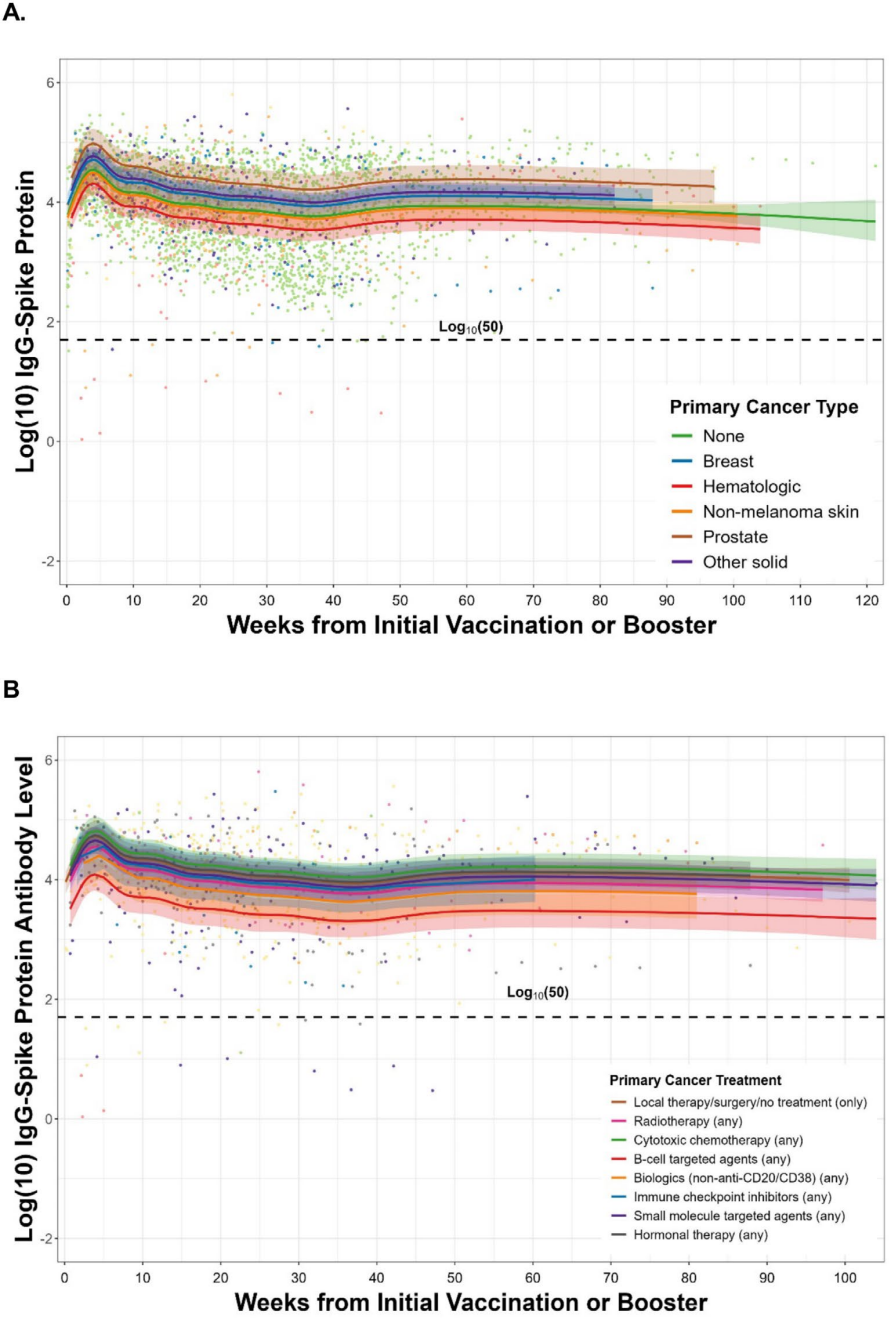
SARS‐CoV‐2 longitudinal IgG‐Spike receptor binding domain (IgG‐SRBD) protein antibody response over time displayed using natural cubic splines, indexed by weeks from their initial vaccine series or last booster, by cancer subtype (Panel A) and by cancer treatment exposures (Panel B).

We used multivariable mixed‐effects linear models to evaluate the association of cancer subtype and cancer treatment exposures with longitudinal IgG‐SRBD response over time. For cancer subtype, hematologic cancers (beta −0.22; *p* = 0.038) were associated with reduced IgG‐SRBD response over time, while prostate cancer (beta 0.44; *p* = 0.001) was associated with increased IgG‐SRBD response over time, in a model accounting for age, sex, race/ethnicity, comorbidities, and SARS‐CoV‐2 infection and vaccination related variables (Table 2, Figure 1A). For cancer treatment exposures, B‐cell targeted agents (beta −0.49; *p* = 0.002) were associated with reduced IgG‐SRBD response over time, while cytotoxic chemotherapy (beta 0.27; *p* = 0.016) and local therapy only (beta 0.18; *p* = 0.030) were associated with increased IgG‐SRBD response over time in a multivariable model accounting for the same demographic, clinical, and SARS‐CoV‐2 related variables (Table 2, Figure 1B).

**TABLE 2:** Association of cancer type and cancer treatment exposures with humoral immune response, represented by longitudinal anti‐spike IgG antibody level.

Outcome: anti‐spike IgG antibody level (IgG‐SRBD)	Beta estimate^a^	SE	*p*	Partial *R* ^2^
**Model A: Cancer subtypes in relation to IgG‐SRBD**				
Metastatic disease	0.10	0.13	0.46	0.001
Cancer subtype				
Breast	0.17	0.09	0.06	0.004
Hematologic	−0.22	0.11	0.038	0.005
Nonmelanoma skin	−0.04	0.10	0.72	0.000
Prostate	0.44	0.14	0.001	0.012
Liver, renal, melanoma, or other solid	0.23	0.10	0.017	0.007
**Model B: Cancer treatment exposures in relation to IgG‐SRBD**				
Metastatic disease	0.11	0.14	0.45	0.001
Cancer treatment				
Local therapy/surgery (only)	0.18	0.08	0.030	0.006
Radiotherapy (any)	−0.02	0.10	0.81	0.000
Cytotoxic chemotherapy (any)	0.27	0.11	0.016	0.005
B‐cell targeted agents (any)	−0.49	0.15	0.002	0.010
Biologics (non‐B‐cell targeted) (any)	−0.15	0.19	0.42	0.001
Immune checkpoint inhibitors (any)	0.03	0.20	0.87	0.000
Small molecule targeted agents (any)	0.09	0.13	0.45	0.001
Hormonal therapy (any)	0.19	0.11	0.10	0.003

^a^ Analyses were performed in participants with at least 1 blood draw with serology assayed, including *N* = 833 participants contributing a total of 4682 blood draw encounters with serology measured at each encounter. Estimates for each model are derived from a multivariable mixed‐effect linear regression with individual‐level repeated measures as random effects, adjusting for all the covariates shown above in addition to ages, sex, race, ethnicity, obesity, hypertension, diabetes, organ transplant status, heart disease, autoimmune disease, liver disease, kidney disease, neurological disease, healthcare employee status, total number of SARS‐CoV‐2 infections, total number of encounter times, number of booster vaccinations prior to a given blood draw, SARS‐CoV‐2 infection preceding first vaccination, log_10_(IgG‐N) level, type of vaccine prior to blood draw, and a multiplicative term representing the interaction between prior booster number and time.

### 3.3 Risk for New‐Onset SARS‐CoV‐2 Omicron and Subvariant Infection

During the follow‐up period, a total of 626 Omicron (and subvariant) infections occurred among 507 participants, with an overall 41.1% (*n* = 423) uninfected rate in the cohort. Healthy participants compared to those with cancer were more likely to have been infected prior to the Omicron era (11.1% vs. 4.4%; *p* = 0.003). For new infections occurring during the Omicron era, there were 170 μm and subvariant infections occurring among the 219 patients with cancer, compared to 456 infections occurring among the 711 healthy participants. In multivariable Poisson regression analyses, hematologic cancer subtype (incidence rate ratio [IRR] 1.64, 95% confidence interval [CI] 1.02–2.59; *p* = 0.037) and exposure to B‐cell targeted agents (IRR 2.08, 95% CI 1.06–3.96; *p* = 0.030) were each associated with an increased number of Omicron and subvariant infections after accounting for demographic, clinical, and SARS‐CoV‐2 related variables, including the total number of booster vaccinations received, any infection prior to vaccination, total number of blood draw encounters, and total study follow‐up time (Table 3).

**TABLE 3:** Association of cancer treatment exposures with risk for new‐onset Omicron and subvariant infections.

Outcome: frequency of new‐onset omicron and subvariant infections	IRR (95% CI)^a^	*p*
**Model A: cancer subtypes in relation to infection risk**		
Metastatic disease	1.09 (0.58, 1.89)	0.77
Cancer subtype		
Breast	1.32 (0.86, 1.99)	0.19
Hematologic	1.64 (1.02, 2.59)	0.037
Nonmelanoma skin	1.15 (0.70, 1.84)	0.57
Prostate	1.26 (0.66, 2.29)	0.47
Liver, renal, melanoma, or other solid	1.20 (0.76, 1.86)	0.42
**Model B: cancer treatments in relation to infection risk**		
Metastatic disease	1.12 (0.58, 1.98)	0.72
Cancer treatment		
Local therapy/surgery (only)	1.10 (0.75, 1.59)	0.62
Radiotherapy (any)	1.02 (0.64, 1.58)	0.95
Cytotoxic chemotherapy (any)	0.76 (0.43, 1.30)	0.32
B‐cell targeted agents (any)	2.08 (1.06, 3.96)	0.030
Biologics (non‐B‐cell targeted) (any)	1.33 (0.56, 2.96)	0.50
Immune checkpoint inhibitors (any)	0.90 (0.25, 2.52)	0.86
Small molecule targeted agents (any)	1.06 (0.61, 1.83)	0.83
Hormonal therapy (any)	1.09 (0.65, 1.77)	0.75

^a^ Analyses were performed in *N* = 1029 participants including 229 with cancer and 800 healthy referent controls under surveillance for new‐onset Omicron infections (defined as occurring after December 15, 2021, through March 10, 2024); the median and IQR of new‐onset infections was 1 [0, 1] in cancer patients and 0 [0, 1] in healthy controls. Estimates for each model are derived from multivariable Poisson regression adjusted for all the covariates shown in addition to age, sex, race, ethnicity, obesity, hypertension, diabetes, organ transplant status, heart disease, autoimmune disease, liver disease, kidney disease, neurological disease, healthcare employee status, total number of encounter times, total number of booster vaccinations in the full follow‐up period, total months of follow‐up length, SARS‐CoV‐2 infection preceding first vaccination. IRR, incidence rate ratio.

### 3.4 Risk for More Severe SARS‐CoV‐2 Omicron and Subvariant Infection

Of all participants studied, including patients who did not experience a new infection during the period of follow‐up, the frequency of moderate severity Omicron and subvariant infection occurring (i.e., association with lower respiratory disease) was 33.2% among those with active or recent cancer treatment compared to 7.2% among healthy control participants (*p* < 0.001). There were no severe or critical illnesses observed. Among the 626 Omicron and subvariant infections, 23.5% were moderate illness, and this rate was significantly increased in patients with active or recent cancer treatment (51.2%) compared to healthy controls (13.2%) (Table 4). In multivariable ordinal logistic regression, all individual subtypes except the other category were significantly associated with an increased risk of moderate Omicron and subvariant infection accounting for demographic, clinical, and SARS‐CoV‐2 variables (all *p* < 0.05, Table 5), though the status of metastatic disease was not (*p* = 0.78). With respect to cancer treatment exposures, B‐cell targeted therapy (OR 4.81; 95% CI 1.78–12.93; *p* = 0.002), small molecule targeted agents (OR 2.34; 95% CI 1.05–5.23; *p* = 0.037), or treatment with local therapy only (OR 1.82; 95% CI 1.05–3.15; *p* = 0.032) were each associated with an increased risk of more severe infection accounting for demographic, clinical, and SARS‐CoV‐2 variables. Results were similar for both cancer subtypes and cancer treatment exposures in analyses restricted to the subset of *N* = 507 participants who experienced any type of new‐onset Omicron and subvariant infection (Table S3).

**TABLE 4:** Detailed distribution and associated severity of SARS‐CoV‐2 infections.

Number of COVID infections (*n* = 817)	Total infections	Cancer	Healthy referent	*p*
	817	197	620	
Infection prior to Omicron era, *n* (%)	191 (23.4)	27 (13.7)	164 (26.5)	< 0.001
Overall infection severity, *n* (%)^a^				< 0.001
Asymptomatic or presymptomatic	256 (31.3)	45 (22.8)	211 (34.0)	
Mild illness	223 (27.3)	38 (19.3)	185 (29.8)	
Moderate illness	147 (18.0)	87 (44.2)	60 (9.7)	
Severe	0 (0.0)	0 (0.0)	0 (0.0)	
Critical	0 (0.0)	0 (0.0)	0 (0.0)	
Infection during Omicron era, *n* (%)	626 (76.6)	170 (86.3)	456 (73.5)	< 0.001
Omicron infection severity, *n* (%)^b^				< 0.001
Asymptomatic or presymptomatic	256 (40.9)	45 (26.5)	211 (46.3)	
Mild illness	223 (35.6)	38 (22.4)	185 (40.6)	
Moderate illness	147 (23.5)	87 (51.2)	60 (13.2)	
Severe	0 (0.0)	0 (0.0)	0 (0.0)	
Critical	0 (0.0)	0 (0.0)	0 (0.0)	

Number of participants (*n* = 1029)	Total participants	Cancer	Healthy referent	*p*
	1029	229	800	
Uninfected, *n* (%)	423 (41.1)	84 (36.7)	339 (42.4)	0.142
Infected prior to Omicron era, *n* (%)	99 (9.6)	10 (4.4)	89 (11.1)	0.003
Overall Omicron infection severity, *n* (%)^c^				< 0.001
Asymptomatic or presymptomatic	172 (16.7)	30 (13.1)	142 (17.8)	
Mild illness	201 (19.5)	29 (12.7)	172 (21.5)	
Moderate illness	134 (13.0)	76 (33.2)	58 (7.2)	
Severe	0 (0.0)	0 (0.0)	0 (0.0)	
Critical	0 (0.0)	0 (0.0)	0 (0.0)	
	930	219	711	
Infected during Omicron era, *n* (%)	507 (49.3)	135 (58.9)	372 (46.5)	0.001
Omicron infection severity, *n* (%)				< 0.001
Asymptomatic or presymptomatic	172 (33.9)	30 (22.2)	142 (38.2)	
Mild illness	201 (39.6)	29 (21.5)	172 (46.2)	
Moderate illness	134 (26.4)	76 (56.3)	58 (15.6)	
Severe	0 (0.0)	0 (0.0)	0 (0.0)	
Critical	0 (0.0)	0 (0.0)	0 (0.0)	

^a^ Overall infection severity includes infections pre and postomicron era.

^b^ Omicron infection severity limited to participants who had an Omicron or subvariant infection.

^c^ Overall Omicron infection severity defined as the severity risk observed in the entire cohort.

**TABLE 5:** Association of cancer treatment exposures with Omicron and subvariant infection severity.

Outcome: Omicron infection occurrence and severity	OR (95% CI)^a^	*P*
**Model A: Cancer types in relation to infection severity**		
Metastatic disease	1.13 (0.48, 2.66)	0.78
Cancer subtype		
Breast	3.00 (1.60, 5.67)	< 0.001
Hematologic	6.75 (3.33, 13.71)	< 0.001
Nonmelanoma skin	2.51 (1.26, 4.94)	0.008
Prostate	5.70 (2.14, 15.15)	< 0.001
Liver, renal, melanoma, or other solid	1.90 (0.99, 3.61)	0.05
**Model B: Cancer treatment in relation to infection severity**		
Metastatic disease	0.94 (0.38, 2.34)	0.90
Cancer treatment		
Local therapy/surgery (only)	1.82 (1.05, 3.15)	0.032
Radiotherapy (any)	1.25 (0.67, 2.31)	0.49
Cytotoxic chemotherapy (any)	0.76 (0.35, 1.67)	0.50
B‐cell targeted agents (any)	4.81 (1.78, 12.93)	0.002
Biologics (non‐B‐cell targeted) (any)	1.05 (0.32, 3.47)	0.93
Immune checkpoint inhibitors (any)	0.91 (0.21, 4.00)	0.91
Small molecule targeted agents (any)	2.34 (1.05, 5.23)	0.037
Hormonal therapy (any)	1.90 (0.92, 3.90)	0.08

^a^ Analyses were performed in *N* = 930 participants including 219 individuals with cancer and 711 healthy referent controls who were under surveillance for new‐onset Omicron infections from December 15, 2021, through March 10, 2024. There were a total of 626 new infection events including 256 asymptomatic or presymptomatic infections, 223 mild infections, and 147 moderate infections. Estimates for each model are derived from a multivariable ordinal logistic regression adjusted for all the covariates shown above in addition to age, sex, race, ethnicity, obesity, hypertension, diabetes, organ transplant status, heart disease, autoimmune disease, liver disease, kidney disease, neurological disease, healthcare employee status, total number of encounter times, total number of booster vaccinations in the full follow‐up period, total months of follow‐up length, and SARS‐CoV‐2 infection preceding the first vaccination.

## 4 Discussion

In a large, longitudinal cohort study involving a diverse population of adults with longitudinal SARS‐CoV‐2 serological assays, infection surveillance data, and comprehensive cancer treatment evaluation, we observed that patients with hematologic cancers or exposed to B‐cell targeted therapy (anti‐CD20/anti‐CD38) had significantly reduced humoral immunity to SARS‐CoV‐2 in addition to more frequent and more severe Omicron and subvariant infections. Furthermore, the presence of most nonhematologic active cancer or treatment with only local therapy (> 75% surgery/local excision) or any small molecule targeted agents was associated with more severe, albeit not more frequent, Omicron infections. Our findings offer insights regarding the types of impaired or dysregulated immunity seen among patients with cancer in response to the SARS‐CoV‐2 Omicron and subvariants circulating in the community. Although there were no observed severe or critical infections, patients with cancer had a 4‐fold increased rate of moderate illness (associated with lower respiratory disease), and so further protective measures, such as passive immunity therapy, may be warranted in these higher risk subgroups.

Our findings extend from prior studies that have shown that cancer treatment exposures may differentially impact the immune response of vaccination or severity of SARS‐CoV‐2 infection [9, 10, 11, 12, 13, 21], although most studies were performed prior to the emergence of Omicron. Amid scant prior Omicron era studies, Mair et al. reported higher rates of breakthrough infections in patients with solid and hematologic cancers treated with anti‐neoplastic systemic therapies, as well as reduced IgG‐SRBD in 26 patients with hematologic cancers treated with B‐cell targeted therapy [22], consistent with our observations. Conversely, Liu et al. found a similar rate of severe/critical Omicron cases (3%) but no impact on severity of Omicron variant infections in relation to recent anti‐cancer treatment [23]; however, this study performed only univariable analysis, was comprised of a higher proportion of patients with lung cancer (31%) and metastatic disease (48%) with palliative intent treatment (50%), and B‐cell directed therapies were not isolated from the ‘targeted therapy’ group. This is an important distinction, given that patients with hematologic cancers remain at increased risk for SARS‐CoV‐2 morbidity and mortality [24, 25, 26], and B‐cell targeted therapies (i.e., anti‐CD20) have been associated with blunted SARS‐CoV‐2 humoral immune responses in the setting of hematologic cancers [27, 28, 29] as well as B‐cell mediated autoimmune disease [30]. Similarly, anti‐CD38 therapy with daratumumab for multiple myeloma has been associated with reduced antibody response to SARS‐CoV‐2 mRNA vaccine [28, 29]. In addition, others have reported that exposure to immune checkpoint inhibitors can increase the risk of severe SARS‐CoV‐2 outcomes [31, 32], though our exposed sample size was small (*n* = 11). Further, we did not observe an impact of radiotherapy on Omicron and subvariant infection outcomes, which is consistent with Liu et al., who reported among 33 patients exposed to chest radiotherapy during the Omicron era no significant association with infection severity. Notably, we did observe that exposure to small molecule targeted agents was associated with an increased risk of greater infection severity, for which this class effect may be driven by a high proportion of proteasome inhibitors (comprised nearly 50%) in this heterogenous cohort, which are known to enhance susceptibility to viral infection [33], and bortezomib‐based therapy in close proximity to SARS‐CoV‐2 infection has been associated with a higher risk of severe disease in the pre‐Omicron era [34, 35]. Lastly, we observed that treatment with local therapy only (> 75% surgery/local excision) was additionally associated with an increased risk of greater infection severity, which may reflect unaccounted for comorbidities and/or risk factors and is worthy of further investigation.

Recognizing that cancer patient populations are intrinsically heterogeneous with respect to their demographic and clinical characteristics, in addition to their treatment exposures, our findings underscore key differentiating aspects of their SARS‐CoV‐2 immunity. We observed, for instance, paradoxically higher or equivalent longitudinal humoral response among participants with prostate or breast cancer, respectively, potentially related to the effects of hormonal therapies on antibody response or occult prior infection or both [15, 36]—though hormonal therapy exposure was not significantly associated with increased risk of infection frequency or severity in this study. These findings are suggestive that these patients benefit from preserved first‐line immune response mechanisms or other forms of immune memory, yet suffer from disrupted second‐line or systemic immune dysregulation that manifests clinically as a persistent risk for more severe SARS‐CoV‐2 infection [37, 38]. In contrast to these patients with solid tumor cancers, participants with hematologic malignancies and especially those receiving B‐cell targeted therapies had both greater frequency and greater risks for more severe infection—underscoring profound immune‐suppression likely involving acute and memory innate and adaptive immunity to SARS‐CoV‐2 and emphasizing the need for administering passive immune therapies including as broadly‐ acting neutralizing antibodies in these patients [39, 40]. Notwithstanding the expected continued shifts in variant susceptibility to passive immunity therapies [41], patients with broad and chronic substantially immunocompromised states may yet derive benefit from enhanced protective strategies and further development of next‐generation neutralizing antibodies is likely warranted [42, 43, 44].

This study has several limitations. First, despite the large size of the primary source cohort (> 10,000 participants), the overall rate of moderate Omicron and subvariant illness was low (13%), with no severe or critical illnesses observed, and the number of patients with active cancer receiving anti‐cancer treatment during the pandemic was modest (*n* = 229). There was a significant 4‐fold increased risk of moderate illness among patients with cancer (vs healthy referent controls), though rates were not compared to comorbidity (noncancer) matched participants and therefore, despite adjustment for confounding variables, may reflect a larger absolute difference in this setting. Finally, the study was conducted among employees and patients at a single health system in a single geographic region, and so further studies are needed to examine the extent to which our findings may be generalizable to other populations.

In conclusion, in this large, longitudinal cohort study we observed that patients with hematologic cancer or exposed to B‐cell targeted agents had significantly reduced humoral immunity, more frequent, and more severe Omicron and subvariant infections. Further, the presence of active breast, prostate, or nonmelanoma skin cancer or treatment with local therapy or small molecular agents was associated with more severe, but not more frequent, Omicron infection. Although there were no severe or critical infections observed, patients with cancer had a 4‐fold increased rate of moderate illness, and further protective measures are likely warranted for these vulnerable individuals.

## Author Contributions

**Katelyn M. Atkins:** conceptualization (equal), data curation (equal), investigation (equal), methodology (equal), supervision (equal), validation (equal), visualization (equal), writing – original draft (equal), writing – review and editing (equal). **Minhao Wang:** formal analysis (equal), investigation (equal), methodology (equal), software (equal), validation (equal), visualization (equal), writing – review and editing (equal). **Katrina D. Silos:** data curation (equal), investigation (equal), methodology (equal), validation (equal). **Sandy Y. Joung:** data curation (equal), formal analysis (equal), investigation (equal), methodology (equal), project administration (equal), resources (equal), validation (equal), writing – review and editing (equal). **Asneh Singh:** data curation (equal), investigation (equal), methodology (equal), validation (equal). **Olivia Peony:** data curation (equal), investigation (equal), methodology (equal), validation (equal). **Jordan O. Gasho:** data curation (equal), investigation (equal), methodology (equal), validation (equal). **Yeran Lee:** data curation (equal), investigation (equal), methodology (equal), validation (equal). **Kenia Gastelum:** data curation (equal), investigation (equal), methodology (equal), validation (equal). **Beatrice Alessandra Filart:** data curation (equal), investigation (equal), methodology (equal), validation (equal). **Alan C. Kwan:** data curation (equal), investigation (equal), methodology (equal), project administration (equal), validation (equal), writing – review and editing (equal). **Marilyn Mendez:** data curation (equal), investigation (equal), methodology (equal), validation (equal). **Yunxian Liu:** conceptualization (equal), data curation (equal), formal analysis (equal), investigation (equal), methodology (equal), software (equal), supervision (equal), validation (equal), visualization (equal), writing – review and editing (equal). **Patrick Belen:** data curation (equal), investigation (equal), methodology (equal), validation (equal). **John C. Prostko:** formal analysis (equal), investigation (equal), methodology (equal), resources (equal), software (equal). **Edwin C. Frias:** formal analysis (equal), investigation (equal), methodology (equal), resources (equal), software (equal). **Joseph E. Ebinger:** data curation (equal), investigation (equal), methodology (equal), project administration (equal), resources (equal), validation (equal), visualization (equal), writing – review and editing (equal). **Sonia Sharma:** conceptualization (equal), funding acquisition (equal), investigation (equal), methodology (equal), project administration (equal), resources (equal), visualization (equal), writing – review and editing (equal). **Kimia Sobhani:** conceptualization (equal), data curation (equal), formal analysis (equal), funding acquisition (equal), investigation (equal), methodology (equal), project administration (equal), resources (equal), software (equal), supervision (equal), validation (equal), visualization (equal), writing – review and editing (equal). **Susan Cheng:** conceptualization (equal), data curation (equal), formal analysis (equal), funding acquisition (equal), investigation (equal), methodology (equal), project administration (equal), resources (equal), software (equal), supervision (equal), validation (equal), visualization (equal), writing – review and editing (equal).

## Disclosure

K.M.A. reports honoraria from OncLive, outside of the submitted work. The remaining authors have nothing to disclose.

## Conflicts of Interest

J.C.P. and E.C.F. work for Abbott Diagnostics, a company that performed the serological assays on the biospecimens that were collected for this study. K.S. has served as a consultant for Abbott Diagnostics. S.Y.J. has served as a consultant for Sapient Bioanalytics, a company that supported the collection and processing of samples for this study. The remaining authors have no relevant potential conflicts.

## Supporting information


Data S1.

## Data Availability

Requests for de‐identified data may be directed to the corresponding authors (K.M.A. and S.C.) and will be reviewed by the Office of Research Administration at Cedars‐Sinai Medical Center prior to issuance of data sharing agreements, which are designed to ensure patient and participant confidentiality.
