# Supplementary material for: Impact of Cancer Subtype and Cancer Therapy Exposures on SARS‐CoV‐2 Outcomes in the Omicron and Subvariant Era
Source: Cancer Med. 2025 Jul 22;14(14):e71082. doi: 10.1002/cam4.71082 (PMC12281020; doi:10.1002/cam4.71082)
Supplement: Supplementary file 1 — Data S1. [file CAM4-14-e71082-s001.docx]

**Impact of Cancer Subtype and Cancer Therapy Exposures on SARS-CoV-2 Outcomes**

**in the Omicron and Subvariant Era**

SUPPLMENTAL MATERIAL

| Supplemental Figure 1 | Pg. 2 |
| --- | --- |
| Supplemental Table 1 | Pg. 3 |
| Supplemental Table 2 | Pg. 4 |
| Supplemental Table 3 | Pg. 7 |
|  |  |

**Correspondence.** Katelyn M. Atkins, MD, PhD, Department of Radiation Oncology, Cedars-Sinai Medical Center, Los Angeles, CA, katelyn.atkins@cshs.org; and, Susan Cheng, MD, MPH, Department of Cardiology, Smidt Heart Institute, Cedars-Sinai Medical Center, Los Angeles, CA, susan.cheng@cshs.org.

**Supplemental Figure 1.**

306 cancer patients having documented cancer type

931 participants having no comorbidity documented and chosen as healthy referent group

10640 participants enrolled in the source cohort

1029 participants included in analyses

(800 referent healthy participants and 229 cancer patients)

177 participants missing key covariates data

833 participants drew at least once and selected for IgG-S analysis

(620 referent healthy participants and 213 cancer patients)

31 cancer patients received primary cancer treatment prior to pandemic

**Supplemental Table 1.** Frequency of types of cancer in the study cohort.

| **Cancer Types** | **Number (%)** |
| --- | --- |
| Non-melanoma skin | 53 (23.1) |
| Breast | 46 (20.1) |
| Hematologic | 41(17.9) |
| Prostate | 22 (9.6) |
| Liver/hepatobiliary | 16 (7) |
| Renal | 7 (3.1) |
| Melanoma | 7 (3.1) |
| Colorectal | 6 (2.6) |
| Lung | 5 (2.2) |
| Small bowel | 4 (1.7) |
| Pancreas | 4 (1.7) |
| Head and Neck | 3 (1.3) |
| Bladder/urinary | 3 (1.3) |
| Uterine | 2 (0.9) |
| Stomach | 2 (0.9) |
| Sarcoma | 2 (0.9) |
| Thymus | 1 (0.4) |
| Testicular | 1 (0.4) |
| Ovarian | 1 (0.4) |
| Vulvar | 1 (0.4) |
| Brain | 1 (0.4) |
| Anal | 1 (0.4) |

**Supplemental Table 2.** Frequency of cancer treatment exposures in the study cohort.

| **Cancer Treatment Types (Any Exposure)** | **Number (%)** |
| --- | --- |
| ***Surgery, local therapies*** | 101 (44.1) |
| Surgery | 143 (62.4) |
| Local excision | 30 (13.1) |
| Active surveillance (prostate cancer) | 14 (6.1) |
| Trans-arterial chemoembolization | 8 (3.5) |
| Ablation | 8 (3.5) |
| Trans urethral resection of bladder tumor | 1 (0.4) |
| High-intensity focused ultrasound | 1 (0.4) |
| Other | 12 (7.1) |
| ***Cytotoxic chemotherapy*** | 57 (24.9) |
| 5-Fluorouracil + irinotecan | 1 (0.4) |
| 5-Fluorouracil or capecitabine | 4 (1.7) |
| 5-fluorouracil or capecitabine, leucovorin, oxaliplatin | 3 (1.3) |
| 5-fluorouracil, leucovorin, oxaliplatin, docetaxel | 1 (0.4) |
| 5-Fluorouracil/capecitabine + mitomycin C | 3 (1.3) |
| Bendamustine | 3 (1.3) |
| Capecitabine + gemcitabine | 1 (0.4) |
| Carboplatin + docetaxel | 1 (0.4) |
| Carboplatin + etoposide | 1 (0.4) |
| Carboplatin + paclitaxel | 3 (1.3) |
| Carboplatin + pemetrexed | 3 (1.3) |
| Cisplatin | 1 (0.4) |
| Cisplatin + gemcitabine | 1 (0.4) |
| Cladribine | 1 (0.4) |
| Cyclophosphamide | 5 (2.2) |
| Cyclophosphamide, doxorubicin hydrochloride, vincristine sulfate, prednisone | 2 (0.9) |
| Cytarabine | 1 (0.4) |
| Dacarbazine + doxorubicin | 1 (0.4) |
| Docetaxel, carboplatin | 4 (1.7) |
| Docetaxel, cyclophosphamide | 2 (0.9) |
| Doxorubicin + cyclophosphamide | 1 (0.4) |
| Doxorubicin, bleomycin sulfate, vinblastine sulfate, dacarbazine | 1 (0.4) |
| Etoposide, prednisone, vincristine, cyclophosphamide, doxorubicin hydrochloride | 1 (0.4) |
| Fludarabine | 1 (0.4) |
| Fludarabine + cyclophosphamide | 2 (0.9) |
| Gemcitabine | 1 (0.4) |
| Gemcitabine + oxaliplatin | 1 (0.4) |
| Gemcitabine + paclitaxel | 3 (1.3) |
| Hydroxyurea | 1 (0.4) |
| Hyperthermic intraperitoneal chemotherapy | 1 (0.4) |
| Lenalidomide | 8 (3.5) |
| Paclitaxel | 2 (0.9) |
| Pomalidomide | 1 (0.4) |
| Stem cell transplant | 10 (4.3) |
| Temozolomide | 2 (0.9) |
| Vincristine sulfate, doxorubicin, cyclophosphamide | 1 (0.4) |
| ***Radiation Therapy (by Technique)*** | 69 (30.1) |
| External beam radiotherapy | 62 (27.1) |
| Brachytherapy | 1 (0.4) |
| Selective internal radiation therapy (yttrium-90) | 6 (2.6) |
| ***Radiation Therapy (by Site)*** | 69 (30.1) |
| Brain | 1 (0.4) |
| Head and neck | 4 (1.7) |
| Breast | 26 (11.4) |
| Thorax | 2 (0.9) |
| Abdomen (liver, pancreas) | 7 (3.1) |
| Pelvis (anal/rectal/genitourinary) | 16 (7.0) |
| Bone | 8 (3.5) |
| Total body irradiation | 3 (1.3) |
| Skin | 1 (0.4) |
| ***Immune Checkpoint Inhibitors*** | 11 (4.8) |
| Avelumab | 1 (0.4) |
| Ipilimumab | 1 (0.4) |
| Nivolumab | 4 (1.7) |
| Pembrolizumab | 5 (2.2) |
| Camrelizumab | 1 (0.4) |
| Cemiplimab | 1 (0.4) |
| ***Small Molecule Targeted Therapy*** | 34 (14.8) |
| Proteasome inhibitors | 16 (7) |
| Bortezomib | 14 (6.1) |
| Carfilzomib | 3 (1.3) |
| Tyrosine kinase inhibitors | 9 (3.9) |
| Ibrutinib | 3 (1.3) |
| Imatinib | 3 (1.3) |
| Apatinib | 1 (0.4) |
| Acalabrutinib | 1 (0.4) |
| Pazopanib | 1 (0.4) |
| B-cell lymphoma 2 (Bcl-2) targeted agent (venetoclax) | 4 (1.7) |
| Somatostatin agonists (octreotide, lanreotide) | 4 (1.7) |
| Neurotrophic tyrosine receptor kinase (NTRK) inhibitor | 2 (0.9) |
| Entrectinib | 1 (0.4) |
| Larotrectinib | 1 (0.4) |
| Vascular Endothelial Growth Factor (VEGF) inhibitor | 1 (0.4) |
| poly-ADP ribose polymerase (PARP) inhibitor (talazoparib) | 1 (0.4) |
| Epidermal growth factor receptor (EGFR) inhibitor (afatinib) | 1 (0.4) |
| Hedgehog pathway inhibitor (vismodegib) | 1 (0.4) |
| ***B-cell targeted therapy*** | 26 (11.4) |
| Anti CD20 | 12 (5.2) |
| Rituximab | 10 (4.4) |
| Obinutuzumab | 2 (0.9) |
| Anti CD38 (daratumumab) | 14 (6.1) |
| ***Biologic (non-B-cell targeted) agents*** | 13 (5.7) |
| Human epidermal growth factor receptor (HER2) inhibitor | 7 (3.1) |
| Trastuzumab | 6 (2.6) |
| Pertuzumab | 1 (0.4) |
| Bevacizumab | 3 (1.3) |
| Blinatumomab | 1 (0.4) |
| Elotzumab | 1 (0.4) |
| Interferon | 1 (0.4) |
| Elranatamab | 1 (0.4) |
| Isatuximab | 1 (0.4) |
| ***Hormonal therapy*** | 38 (16.6) |
| Aromatase inhibitors | 19 (8.3) |
| Anastrozole | 11 (4.8) |
| Letrozole | 7 (3.1) |
| Exemestane | 1 (0.4) |
| Tamoxifen | 10 (4.4) |
| Leuprolide | 9 (3.9) |
| Relugolix | 2 (0.9) |
| Bicalutamide | 1 (0.4) |
| Abiraterone acetate | 1 (0.4) |
| ***Combined Modality Treatment*** | |
| Surgery + chemotherapy + RT | 12 (5.2) |
| Chemotherapy + RT | 10 (4.4) |
| Surgery + RT | 9 (3.9) |
| Surgery + chemotherapy | 7 (3.1) |
| Chemotherapy + RT + ICI | 3 (1.3) |

Abbreviations: RT, radiotherapy; ICI, immune checkpoint inhibitor.

**Supplemental Table 3.** Association of cancer treatment exposures with Omicron infection severity, among only participants who developed any Omicron infection (n=507).

| **Outcome: Omicron infection occurrence and severity** | **OR (95% CI)*** | **P value** |
| --- | --- | --- |
| ***Model A: Cancer subtypes in relation to infection severity*** |  |  |
| Metastatic disease | 1.13 (0.36, 3.51) | 0.84 |
| Cancer subtype |  |  |
| Breast | 3.25 (1.48, 7.21) | **0.003** |
| Hematologic | 7.10 (2.83, 17.77) | **<0.001** |
| Non-melanoma skin | 5.21 (2.07, 13.07) | **<0.001** |
| Prostate | 6.11 (1.77, 20.93) | **0.004** |
| Liver, renal, melanoma, or other solid | 1.99 (0.86, 4.68) | 0.11 |
| ***Model B: Cancer treatment in relation to infection severity*** |  |  |
| Metastatic disease | 0.63 (0.19, 2.07) | 0.45 |
| Cancer treatment |  |  |
| Local therapy/surgery (only) | 1.92 (0.95, 3.86) | 0.07 |
| Radiotherapy (any) | 1.75 (0.72, 4.24) | 0.22 |
| Cytotoxic chemotherapy (any) | 1.36 (0.47, 3.96) | 0.58 |
| B-cell targeted agents (any) | 0.96 (0.29, 3.19) | 0.95 |
| Biologics (non-B-cell targeted) (any) | 1.40 (0.26, 7.42) | 0.69 |
| Immune checkpoint inhibitors (any) | 6.75 (0.45, 101.67) | 0.17 |
| Small molecule targeted agents (any) | 5.42 (1.87, 15.89) | **0.002** |
| Hormonal therapy (any) | 2.08 (0.76, 5.71) | 0.15 |

*Analyses were performed in N=507 participants including 135 individuals with cancer and 372 healthy referent controls who were under surveillance for new-onset Omicron infections from December 15, 2021 through March 10, 2024. There were a total of 626 new infection events including 256 asymptomatic or presymptomatic infections, 223 mild infections and 147 moderate infections. Estimates for each model are derived from a multivariable ordinal logistic regression adjusted for all the covariates shown above in addition to age, sex, race, ethnicity, obesity, hypertension, diabetes, organ transplant status, heart disease, autoimmune disease, liver disease, kidney disease, neurological disease, healthcare employee status, total number of encounter times, total number of booster vaccinations in full follow-up period, total months of follow-up length, SARS-CoV-2 infection preceding first vaccination.
